# Chromosome-level assembly and analysis of *Camelina neglecta*: a novel diploid model for Camelina biotechnology research

**DOI:** 10.1186/s13068-024-02466-9

**Published:** 2024-01-31

**Authors:** Shuo Wang, Rostislav Y. Blume, Zhi-Wei Zhou, Shaoping Lu, Tara J. Nazarenus, Yaroslav B. Blume, Weibo Xie, Edgar B. Cahoon, Ling-Ling Chen, Liang Guo

**Affiliations:** 1 National Key Laboratory of Crop Genetic Improvement, Huazhong Agricultural University, Wuhan, China; 2 Institute of Food Biotechnology and Genomics, National Academy of Sciences of Ukraine, Kiev, Ukraine; 3 Center for Plant Science Innovation and Department of Biochemistry, University of Nebraska-Lincoln, Lincoln, USA; 4 College of Life Science and Technology, Guangxi University, Nanning, China; 5 Hubei Hongshan Laboratory, Wuhan, China; 6 Shenzhen Institute of Nutrition and Health, Huazhong Agricultural University, Wuhan, China; 7 Shenzhen Branch, Guangdong Laboratory for Lingnan Modern Agriculture, Genome Analysis Laboratory of the Ministry of Agriculture, Agricultural Genomics Institute at Shenzhen, Chinese Academy of Agricultural Sciences, Shenzhen, China

**Keywords:** *Camelina neglecta*, *Camelina sativa*, Synteny analysis, *Camelina* evolution, Oil biosynthesis pathway, Medium-chain fatty acid

## Abstract

*Camelina neglecta* is a new diploid Brassicaceae species, which has great research value because of its close relationship with the hexaploid oilseed crop *Camelina sativa*. Here, we report a chromosome-level assembly of *C. neglecta* with a total length of 210 Mb. By adopting PacBio sequencing and Hi-C technology, the *C. neglecta* genome was assembled into 6 chromosomes with scaffold N50 of 29.62 Mb. *C. neglecta* has undergone the whole-genome triplication (γ) shared among eudicots and two whole-genome duplications (α and β) shared by crucifers, but it has not undergone a specific whole-genome duplication event. By synteny analysis between *C. neglecta* and *C. sativa*, we successfully used the method of calculating Ks to distinguish the three subgenomes of *C. sativa* and determined that *C. neglecta* was closest to the first subgenome (SG1) of *C. sativa*. Further, transcriptomic analysis revealed the key genes associated with seed oil biosynthesis and its transcriptional regulation, including *SAD*, *FAD2*, *FAD3*, *FAE1*, *ABI3*, *WRI1* and *FUS3* displaying high expression levels in *C. neglecta* seeds*.* The high representability of *C. neglecta* as a model species for *Camelina-*based biotechnology research has been demonstrated for the first time. In particular, floral *Agrobacterium tumefaciens* infiltration-based transformation of *C. neglecta*, leading to overexpression of *CvLPAT2*, *CpDGAT1* and *CvFatB1* transgenes, was demonstrated for medium-chain fatty acid accumulation in *C. neglecta* seed oil. This study provides an important genomic resource and establishes *C. neglecta* as a new model for oilseed biotechnology research.

**Supplementary Information:**

The online version contains supplementary material available at 10.1186/s13068-024-02466-9.

## Introduction

False flax, or gold-of-pleasure (*Camelina sativa*), is one of the oldest oil crops in Brassicaceae family, originating from the region of Eastern Europe–Western Asia [61, 73]. During the past decades, this species has attracted strong research interest as an important and promising biofuel crop [5, 69]. In particular, *C. sativa* is viewed as an emerging platform for genetic engineering and gene editing improvement, aiming at producing seed oil with designed fatty acid composition [29, 30, 68]. Close genetic relation of this crop to the widely known model plant, *Arabidopsis thaliana*, high amenability for transformation, facultative self-pollinating nature of this crop and short vegetation cycle make *C. sativa* an ideal candidate for cutting-edge biotechnology research [28, 39, 67].

*C. sativa* research is partially complicated by its allohexaploid nature and limited genetic diversity [33, 44]. Because of this, other representatives of the *Camelina* genus, especially diploids, have received increased attention for genus evolution, genomics and biotechnology research [15]. The recent description and characterization of a new diploid species *Camelina neglecta* significantly enhanced the understanding of evolutionary events that has clarified the origin of the complex hexaploid *C. sativa* species [8]. It has been shown that *C. neglecta* directly contributes to the origin of the first subgenome of *C. sativa* and is also believed to be the progenitor of the second subgenome [46, 72]. In addition, *C. neglecta* has the smallest genome within the *Camelina* genus [15]. Moreover, the importance of *C. neglecta* was further demonstrated by showing its relation to the origin of two other tetraploid *Camelina* representatives—*Camelina rumelica* and tetraploid *Camelina microcarpa* (known as *Camelina intermedia*), making *C. neglecta* a progenitor of all known polyploids within the genus [15, 46, 48]. In addition, recent research suggests that *C. neglecta* is a maternal progenitor of the widely used oilseed *C. sativa* and its closest wild relatives *C. microcarpa* and *C. intermedia*, since they all have inherited cytoplasm directly from *C. neglecta* [9, 45].

These limitations of conventional *C. sativa* breeding raised questions about alternate pathways for its improvement, among which are often discussed `re-synthesis` of this hexaploid crop [46] and genetic engineering techniques [28]. Both approaches require a comprehensive understanding of the genome organization of this crop and its wild progenitors, which participated in the genus evolution. Additionally, genetic manipulations with *C. sativa* require additional efforts for obtaining pure homozygous lines, because of the hexaploid nature of this species. A possible solution for this problem could be the use of diploid *Camelina* representatives for rapid testing of outcomes from genetic manipulations. In this regard, C. *neglecta* seems to be an ideal candidate, since other know diploid *Camelina* species (*C. hispida* and *C. laxa*) are self-incompatible [71]. Moreover, *C. neglecta* has very similar fatty acid composition of seed oil to *C. sativa* [10], making this diploid even more attractive for such investigations.

Furthermore, *Camelina* genomics have been in focus due to its potential to serve as a model for studying mechanisms of plant polyploidy [46]. Attractiveness, of this approach, in particular, grounds on the fact that diploid *Camelina* species (as well as other representatives of Lineage I of Brassicaceae) have not faced whole-genome triplication specific to *Brassica* species from Lineage II [15, 46, 48]. Moreover, *Camelina* polyploid species complex mainly comprised neopolyploids, which usually still retain subgenomes structure of their parental species and still undergo processes of genome fractioning and subgenome dominance, including elimination of gene duplicates and balancing of their expression [16]. To the date, several attempts for *C. neglecta* genome sequencing have been made using different approaches [16, 48], as well as several investigations were made to uncover the genome structural changes within different *Camelina* representatives [15, 46].

In this study, we aimed to (1) produce a high-quality chromosome-level assembly of *C. neglecta*, (2) use comparative genomics analysis to reveal the evolutionary events that shaped the modern genome of *C. neglecta*, and (3) show the role of this species in the evolution of economically important oilseed crop *C. sativa*. In addition, we show for the first time the possibility to use *C. neglecta* as an effective model species for *Camelina* biotechnology research. This was achieved by successful demonstration of an *Agrobacterium tumefaciens*-based floral infiltration transformation protocol and by conducting a comprehensive transcriptomic analysis of key genes associated with *C. neglecta* seed oil content and fatty acid quality.

## Results

### High-quality genome assembly and annotation

**Fig. 1:**
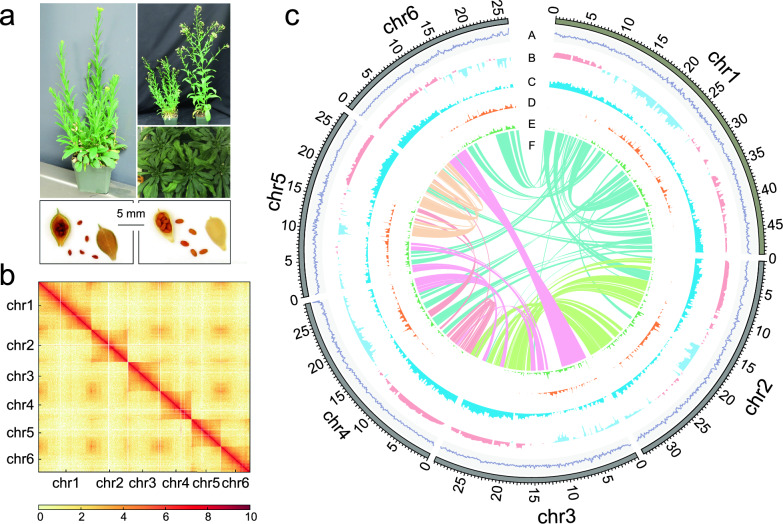
Genome assembly and genomic features of *C. neglecta*. **a** General view of the greenhouse-grown *C. neglecta*: the upper left is the *C. neglecta* during flowering. The upper right is the comparison of *C. neglecta* (left) and *C. sativa cv. Suneson* (right) during the beginning of seeds ripening. The middle of right is the leaf rosette of *C. neglecta* before vernalization. The bottom is the general view of opened seed pods and seeds of *C. neglecta* (left) and *C. sativa* (right). Bars, 5 mm. **b** Genome-wide Hi-C contact matrix of *C. neglecta* genome. The color intensity represents the frequency of contact between two 50 kb loci. **c** Circos plot of the multidimensional topography for *C. neglecta* genome (window size of 100 Kb). From outer to inner represented GC content (A), A/B compartment (B), gene density (C), LTR/Gypsy density (D), LTR/Copia density (E), collinear links (F)

*C. neglecta* contributes to the first subgenome of *C. sativa* and it generally has smaller plant size than *C. sativa* (Fig. 1a). A genome survey based on 17-mer frequency revealed that the genome size of *C. neglecta* is 255.04 Mb, which is comparable to previous assessments using flow cytometry (Additional file 1: Fig. S1) [48]. The genome of *C. neglecta* was assembled using multifaceted sequencing approaches, including 149.74 Gb PacBio long reads (~ 714 ×) for de novo assembly, 58.96 Gb Illumina pair-end reads (~ 281 ×) for genome correction, and 52.54 Gb high-throughput chromosome conformation capture (Hi-C) reads (~ 250 ×) for genome assembly (Additional file 2: Table S1). Through these approaches, we generated a 210 Mb chromosome-level *C. neglecta* genome assembly with 238 contigs and an N50 size of 11.77 Mb (Table 1). These contigs were anchored into 6 chromosome-level pseudochromosomes (2*n* = 12) with the scaffold N50 of 29.62 Mb using Hi-C reads (Fig. 1b). The total pseudochromosome length was 193.9 Mb, with the sizes of pseudochromosome ranging from 26.3 to 48.6 Mb (Additional file 2: Table S2). To assess the accuracy and completeness of the genome assembly, the Illumina pair-end reads were mapped to the assembled genome with the alignment rate of 99.73% and different tissues of RNA sequencing data were also mapped to the assembly with an average alignment rate of 93.01% (Table 1, Additional file 2: Table S3). The Benchmarking Universal Single-Copy Orthologs (BUSCO) assessment implied that 99.1% core eukaryotic genes were captured in the genome assembly (Additional file 2: Table S4). Furthermore, long-terminal repeat retrotransposons assembly index (LAI) reached 18.54, suggesting that the continuity of the assembly meets the criteria for the high-quality reference genome (Table 1). Overall, all assessments suggest a high-quality of the *C. neglecta* genome assembly.

**Table 1:** Statistics of genome assembly and annotation of *C. neglecta*

	Genomic feature	*C. neglecta*
Assembly	Total length (bp)	209,796,048
	Total contig number	238
	Max contig length (bp)	19,403,321
	Contig N50 (bp)	11,768,714
	Number N50	7
	Anchored length (bp)	193,908,758
	Anchored rate	92.4%
	Scaffold N50 (bp)	29,623,700
Assessment	NGS mapping rate	99.73%
	Complete BUSCOs	99.10%
	LAI score	18.54
Annotation	Repetitive sequences (%)	45.30%
	Protein-coding genes	26,595

In total, 26,595 protein-coding genes were annotated in the *C. neglecta* genome through a combination of ab initio, homology-based analyses and RNA sequencing-assisted prediction with mean CDs length of 1238 bp (Additional file 2: Table S5). Among these genes, 99% were functionally annotated according to the results of mapping these genes to multiple protein databases (Additional file 2: Table S6). Transposable elements (TEs) accounted for 45.30% of the estimated *C. neglecta* genome, and long-terminal retrotransposon (LTR) formed the most abundant category of TEs, with LTR/Copia and LTR/Gypsy occupying 8.21% and 16.49% of the genome (Fig. 1c, Additional file 2: Table S7). Estimation of LTR insertion time revealed that the burst of retrotransposon multiplication in *C. neglecta* happened about 1.5–2.0 million years ago (Mya) (Additional file 2: Table S8). In addition, 8,629 non-coding RNAs were predicted in the genome, containing 4,771 ribosomal RNAs, 2,436 transfer RNAs, 126 microRNAs and 1296 small nuclear RNAs (Additional file 2: Table S9).

### Phylogenetic analysis and whole-genome duplication events in C. neglecta

**Fig. 2:**
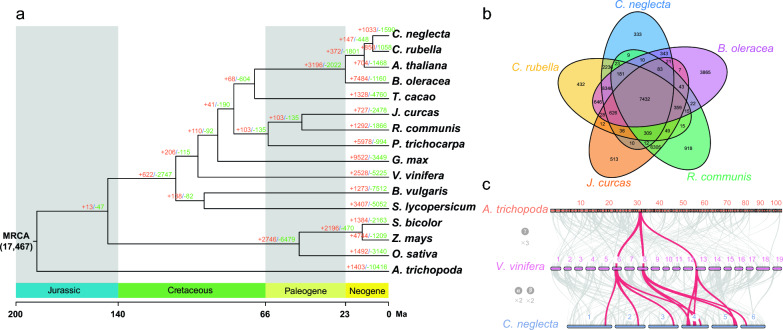
Evolutionary comparison and gene conservation of *C. neglecta* genome. **a** The phylogenetic tree and expansion/contraction of gene families among 16 plant species. The phylogenetic relationship and divergence time estimation is based on all single-copy orthologous gene families shared by selected species. The number at the root (17,467) denotes the total number of gene families predicted in the most recent common ancestor (MRCA). A total of 1,033 gene families are substantially expanded, and 1,590 gene families are contracted in *C. neglecta* compared with other genomes. **b** Shared gene families among *C. neglecta*, *B. oleracea*, *R. communis*, *J. curcas* and *C. rubella*. The five species contain 7,432 common gene families, and *C. neglecta* has 333 specific gene families. **c** Macrosynteny relationship between *C. neglecta*, *V. vinifera*, and *A. trichopoda* genomes. Macrosynteny patterns between *A. trichopoda* and *V. vinifera* show that each *A. trichopoda* region aligns to three syntenic regions in *V. vinifera*, and each *V. vinifera* region aligns to four syntenic regions in *C. neglecta*, which experienced two additional rounds of crucifer genome duplication

A phylogenetic tree was constructed using 174 single-copy orthologous genes in *C. neglecta* and 15 other plant genomes. The results reveal that *C. neglecta* is closely related to the Brassicaceae *Capsella rubella*, and approximately 8.5 Mya, *C. neglecta* diverged from *C. rubella* (Fig. 2a). Moreover, 1033 gene families are expanded and 1590 gene families are contracted in *C. neglecta* genome. Comparing *C. neglecta* with *C. rubella*, *Jatropha curcas*, *Ricinus communis* and *Brassica oleracea*, the 26,595 predicted protein-coding genes in *C. neglecta* genome were clustered into 20,010 gene families, and 7432 gene families are shared by five genomes (Fig. 2b). A total of 118 significant GO teams were enriched in 333 *C. neglecta* unique gene families including “peptide biosynthetic process”, “ATP hydrolysis coupled proton transport”, “NADH dehydrogenase activity”, etc. (Additional file 2: Table S10). To identify the whole-genome duplication events in *C. neglecta,* we compared its genome with the genomes of *Amborella trichopoda*, the most basal lineage of angiosperms and *Vitis vinifera*, an ancient dicotyledonous plant without genome duplication. *C. neglecta* was found to share 12:1 relationship with *A. trichopoda* and 4:1 or 12:3 relationship with *V. vinifera*. This is consistent with *C. neglecta* having undergone two genome duplications (α and β) shared by the Brassicaceae, but no exclusive whole-genome duplication event was found in *C. neglecta* (Fig. 2c).

### Comparative analysis between C. neglecta and hexaploid C. sativa

**Fig. 3:**
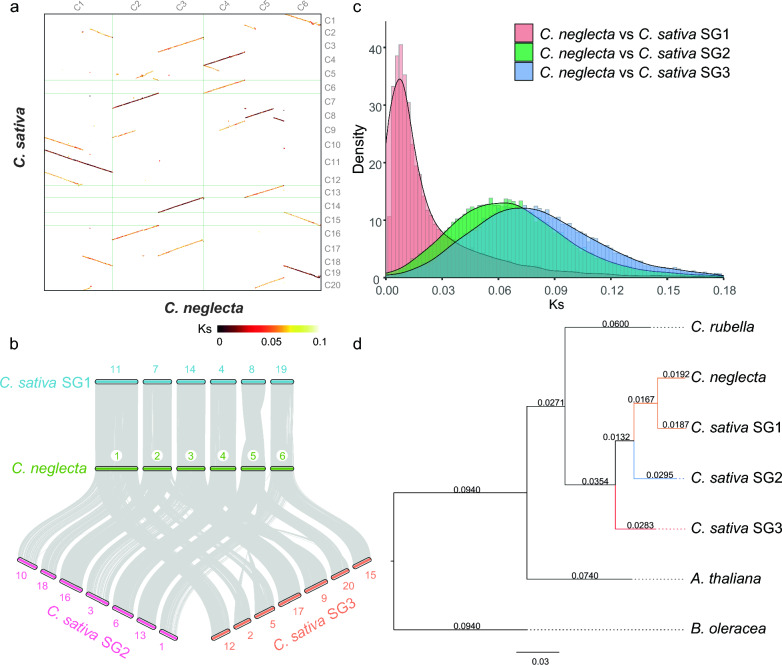
Comparison analysis between *C. neglecta* and hexaploidy *C. sativa*. **a** Collinear synteny blocks between *C. neglecta* and *C. sativa* genomes. Synonymous substitutions rate (Ks) from dark to light indicates that genetic divergence from small to large. **b** Macrosynteny relationship between *C. neglecta* and *C. sativa* subgenomes. **c** Density distributions of the Ks values for homologous genes between *C. neglecta* and *C. sativa* subgenomes. **d** The phylogenetic tree of *C. neglecta*, *C. sativa* subgenomes and other closely related plants. The phylogenetic tree is constructed on the basis of 200 single-copy orthologous genes. The values on the branch are the substitutions between species and the nearest ancestor

Comparison analysis shows that the genome of *C. neglecta* is closest to the first subgenome (SG1) of *C. sativa*. First, we compared the collinearity between *C. neglecta* and *C. sativa* genomes and found that *C. neglecta* and *C. sativa* genome were highly conserved and showed a 1:3 correspondence of collinear synteny blocks. The smallest Ks value was found in one of the three collinear synteny blocks corresponding to each chromosome of *C. neglecta*, indicating that chromosomal segments in this block were the least genetically distinct, while the remaining two blocks were not significantly different (Fig. 3a). To distinguish three subgenomes of *C. sativa*, the collinear synteny blocks were first divided into sub1, sub2 and sub3, and the Ks values of the collinear synteny blocks in each chromosome were calculated (Additional file 1: Fig. S2). The peak of the density distribution of Ks values shows that SG1 of *C. sativa* consists of Cs11, Cs7, Cs14, Cs4, Cs8, Cs19, the second subgenome (SG2) of *C. sativa* consists of Cs10, Cs18, Cs16, Cs3, Cs6, Cs13, Cs1, and the third subgenome (SG3) of *C. sativa* consists of Cs12, Cs2, Cs20, Cs5, Cs9, Cs17, Cs15, which is in agreement with previous studies [15] (Additional file 1: Fig. S3). In addition, the *C. neglecta* and *C. sativa* subgenomes have good collinearity (Fig. 3b), and the density distribution of Ks values for homologous genes shows that SG1 of *C. sativa* and *C. neglecta* have the smallest peak at approximately 0.02, which also indicates that *C. neglecta* is closest to SG1 of *C. sativa* compared to SG2 and SG3 of *C. sativa* (Fig. 3c). Furthermore, a phylogenetic tree constructed using 200 single-copy orthologous genes selected from *A. thaliana*, *B. oleracea*, *C. neglecta*, *C. rubella* and three subgenomes of *C. sativa* also indicates that *C. neglecta* is closest to SG1 of *C. sativa* (Fig. 3d).

### Transcriptomic analysis of key genes in seed oil biosynthesis

**Fig. 4:**
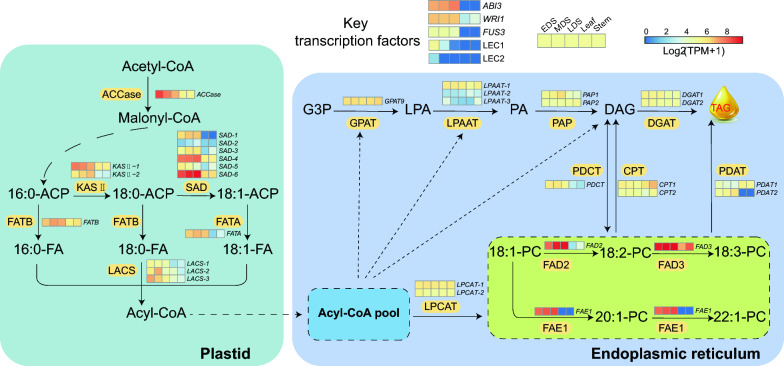
Transcriptomic analysis of *C. neglecta* oil biosynthesis pathway. Simplified diagram of the oil biosynthesis pathway in *C. neglecta*. The heatmaps show the key enzyme-coding genes and transcription factor-coding genes and their expression in different tissues. Based on the fact that oil synthesis genes are highly expressed in seeds, some genes that are low or not expressed in seeds were omitted, including six out of nine copies of *LACS*, one out of two copies of *PDCT* and one out of two copies of *FAE1*. EDS, early developing seed; MDS, mid developing seed; LDS, late developing seed

*C. sativa* is recognized as an important oilseed crop [73]. Additionally, the genome of *C. neglecta* has been found to be highly similar to SG1 of *C. sativa* [15]*,* and their seed oils are both rich in unsaturated fatty acids. To analyze the genetic background for the active accumulation of unsaturated fatty acid in *C. neglecta* seeds, we obtained transcriptomic data from different tissues, including seeds at different developmental stages (early developing seed, mid developing seed and late developing seed), leaf and stem. We investigated the key genes involved in the oil biosynthesis pathway in seed (Fig. 4), and found that most of them were highly expressed in seeds, but low or even unexpressed in leaf and stem (Additional file 2: Table S11). Notably, several key desaturase genes were identified in lipid synthesis such as *SAD* (key desaturase catalyzing the conversion of 18:0-ACP to 18:1-ACP in the plastid), *FAD2* (fatty acid desaturase 2 desaturating 18:1-PC to 18:2-PC in the endoplasmic reticulum), *FAD3* (fatty acid desaturase 3 desaturating 18:2-PC to 18:3-PC in the endoplasmic reticulum) were all highly expressed in seed, explaining the enrichment of unsaturated fatty acids in *C. neglecta* seed. Moreover, *FAE1* (fatty acid elongase 1) encoding a 3-ketoacyl-CoA synthase that initiates the ER fatty acid elongation to produce C20 and C22 very long-chain fatty acids [49] was also found to be highly expressed in seed, which may be the reason why *C. neglecta* is also enriched in very long chain unsaturated fatty acid. In addition to these genes, we also identified a variety of transcription factors involved in the oil biosynthesis pathway such as *abscisic acid insensitive 3* (*ABI3*), *wrinkled 1* (*WRI1*) and *fusca 3* (*FUS3*) which were also highly expressed in seed. Leafy cotyledon 1 (*LEC1*) is mainly expressed in early development seed and Leafy cotyledon 2 (*LEC2*) has very low expression during seed development (Fig. 4). These transcription factors also play an important role in the synthesis and accumulation of seed oil in *C. neglecta.* In summary, the high expression of key genes and transcription factors involved in seed oil biosynthesis appears to contribute to the high oil content of *C. neglecta* and its fatty acid composition.

### Demonstration of C. neglecta as a tool for oilseed biotechnology

**Fig. 5:**
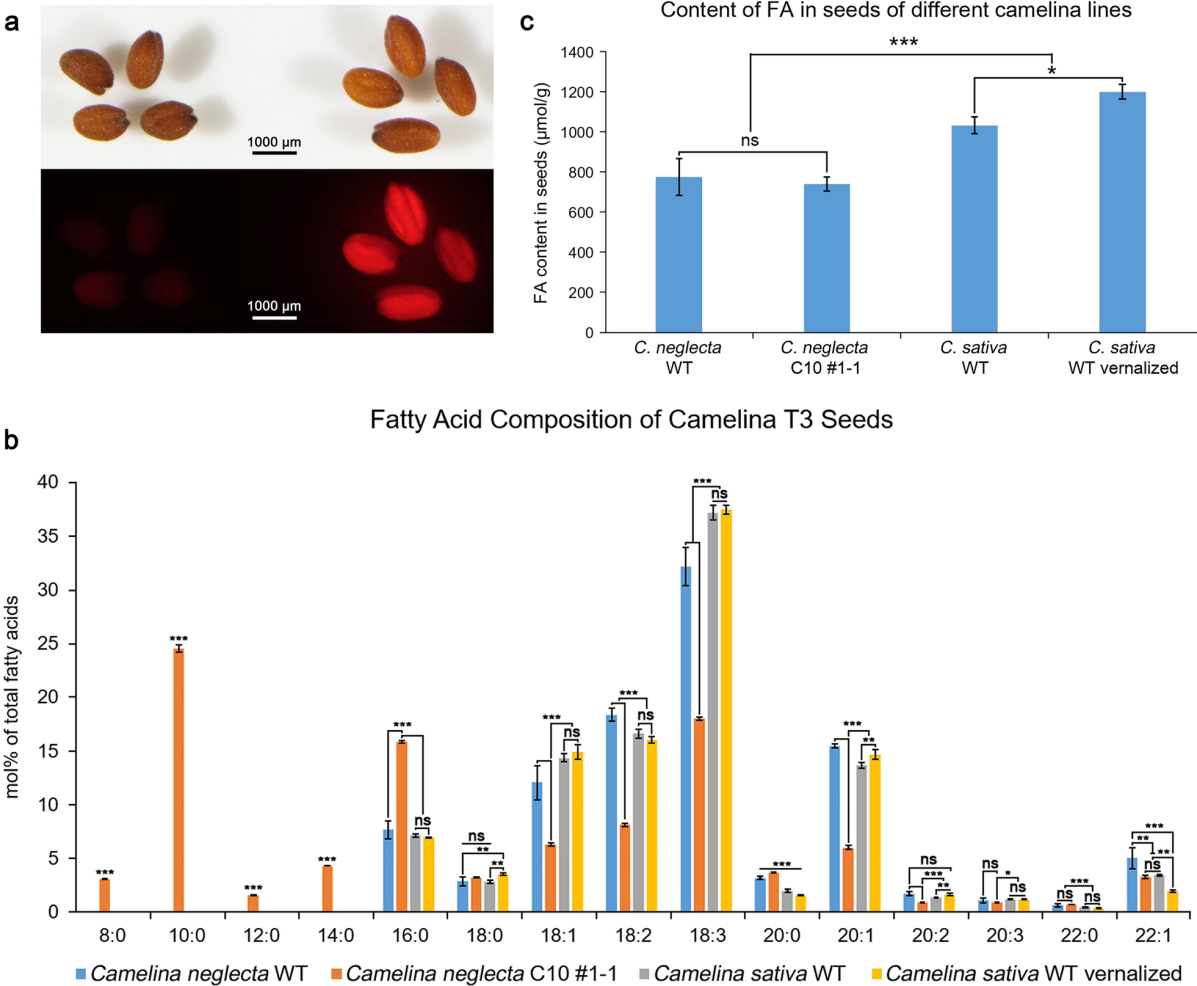
Genetic transformation of *C. neglecta* for biotechnological development of novel seed oils. **a** Differences of the appearance of *C. neglecta* seeds: WT (left) and transformants (right). Upper row—regular view of the seeds, bottom row—visualization of DsRed fluorescence in T_3_ seeds. **b** Fatty acid composition (mol%) of seed oil of the transformed and WT *C. neglecta* lines, as well as of *C. sativa* cv. Suneson, grown without and with vernalization. **c** Total content of fatty acids in seeds (expressed as μmol/g) of the transformed and WT *C. neglecta* lines and vernalized/non-vernalized *C. sativa*. Different level of statistical significance of the investigated parameters is denoted as: *ns* non-significant (*p* > 0.05); *Difference is significant at *p* < 0.05; **Significant at *p* < 0.01; ***Significant at *p* < 0.001

We next examined the amenability of *C. neglecta* for *Agrobacterium tumefaciens* transformation. A vector, containing *CvLPAT2*, *CpDGAT1* and *CvFatB1*, was introduced into *C. neglecta* plants in the same genetic background as used here for genome sequencing. For these studies, we used a floral infiltration protocol similar to the method described for *C. sativa* [40]. Our binary vector backbone pBinGlyRed3 contained a constitutively expressed *Discosoma* sp. red fluorescent protein (DsRed) marker for selection of transgenic seeds based on fluorescence [51]. The transgenes were expressed in *C. neglecta* under the control of strong seed-specific promoters. DsRed-positive seeds obtained from independently produced transgenic lines in T_2_ generation were screened for presence of the transgenes and were further selected to obtain homozygous lines in T_3_ generation (Fig. 5a). The transgene combination used here was previously used for *C. sativa* seed oil modification [29]. In these prior experiments, expression of the transgene combination conferred C10:0 accumulation in *C. sativa* seed to ≤ 24 mol% of fatty acids. Here, a similar effect was observed in T_3_ generation of *C. neglecta* transformants. Seeds from this generation accumulated C10:0 to 25 mol% of total fatty acids (Fig. 5b). No C10:0 was detected in seeds of non-transformed plants. We also detected the accumulation of other medium-chain fatty acids: C8:0 accumulated to 3 mol%, C12:0 accumulated to 1.5 mol%, and C14:0 accumulated to 4.26 mol% of seed total fatty acids. These fatty acids were absent from non-transgenic *C. neglecta* or *C. sativa* seeds. These production of medium-chain fatty acids in the transgenic DsRed-positive seeds was accompanied by increases in C16:0 and large reductions in the polyunsaturated fatty acids linoleic (C18:2) and linolenic (C18:3) acids (Fig. 5b). Notably, the total fatty acid concentration of seeds from the C10 lines calculated on a molar basis was not significantly different from concentrations in seeds from non-transformed *C. neglecta* plants (Fig. 5c).

Given that *C. neglecta*, in contrast to *C. sativa*, has a prolonged life cycle and requires vernalization for flowering, we examined whether these different growth conditions affect the fatty acid profile of *C. sativa* seed oil. It was found that the cultivation *C. sativa* plants with vernalization (similar to *C. neglecta*) has little effect on the composition of C16 and C18 fatty acids in the seed oil. However, vernalization was found to result in a significant decrease in erucic acid (C22:1) and a corresponding increase in gondoic acid (C20:1) compared to seed oil from *C. sativa* plants not exposed to vernalization (Fig. 5b). In addition, seeds from vernalized *C. sativa* plants had ~ 10% higher fatty acid content than those from non-vernalized plants (Fig. 5c).

Comparisons of the total fatty acid concentrations of seeds from these two *Camelina* species revealed a ~ 25% to 35% lower fatty acid content in *C. neglecta* seeds relative to seeds from non-vernalized or vernalized *C. sativa* plants (Fig. 5c). The major difference in fatty acid composition in seeds of these two *Camelina* species was a ~ 15% lower relative amount of linolenic acid (C18:3) and a corresponding increase in the total relative amount of C20:0, C20:1 and C22:1 in seeds from *C. neglecta* plants compared to amounts of these fatty acids in *C. sativa* seeds (Fig. 5b).

## Discussion

*C. sativa* is a widely recognized oilseed, known for its high fatty acid content that renders it a valuable vegetable oil feedstock for food, industrial raw materials, and liquid biofuels, including renewable diesel and sustainable aviation fuel [73]. *C. neglecta* has been reported to be highly similar to SG1 of *C. sativa* and highly collinear to SG2 [15, 46]. In this study, we reported a high-quality genome assembly of *C. neglecta* at the chromosome level. Although two genomes of *C. neglecta* have been previously reported, they exhibited high collinearity (Additional file 1: Figs. S4 and S5) [16, 48]. However, the size of our assembled genome was 3.1 Mb larger than that of Chaudhary et al. at the chromosome level, and is comparable to the assembly of Martin et al. (Additional file 2: Table S12) [16, 48]. Interestingly, an inversion spanning 206,933 bp on chromosome 2 was observed in comparison to the assembly of Chaudhary et al., we verified the accuracy of our assembly by visualizing the PacBio reads at both ends of the breakpoint in this region by IGV (Additional file 1: Fig. S6). We observed a 4.2 Mb inversion on chromosome 2 when comparing the collinearity with the assembly of Martin et al. which was also reported in Chaudhary et al. (Additional file 1: Figs. S4 and S7) [16, 48]. We verified the accuracy of our assembly though Hi-C data. In addition, we found 9 large presence variations in our assembled genome. We checked the PacBio reads of the breakpoints at both ends of these 9 regions, and found that reads at both ends of the breakpoints were evenly spanned, indicating that the results of our assembly are accurate (Additional file 1: Fig. S8).

Notably, the number of protein-coding genes annotated in this study is less than that annotated by Chaudhary et al. (26,595 vs 34,061) [16]. With the help of TransDecoder software, we find that 26,196 of the genes annotated in this study are complete, 3 genes are missing the 5ʹ end, 2 genes have only the middle part of the gene, and 394 genes are incorrectly annotated, giving an accuracy of gene annotation of 98.50%. Among the genes annotated by Chaudhary et al., 29,546 genes are complete, 2,698 genes lacked 5’ end, 273 genes lacked 3ʹ end, 109 genes have only the middle part of the gene, 1435 genes are incorrectly annotated, and the accuracy of gene annotation is 86.74% (Additional file 2: Table S13). The accuracy of our annotated genes is higher than that of Chaudhary et al. [16]. We noted that Chaudhary et al. used the BRAKER software to annotate genes [16]. There are a number of issues mentioned on GitHub for the software which annotates a high number of genes (#513, #541, #522, etc.), probably due to the fact that fewer genome repetitive regions are masked while annotating, leading to a lot of false-positive genes being annotated. Currently, protein-coding gene annotation is mainly based on homology prediction, de novo prediction, and RNA-Seq assisted prediction [7, 25]. Despite the existence of numerous algorithms and tools for gene prediction and annotation [11, 25–27, 34, 43, 54, 58], the accuracy of predictions requires further improvement. The prediction process is complex and involves the integration of multiple algorithms and tools for analysis and validation. Thus, it is imperative to continuously enhance and optimize annotation methods to achieve improved accuracy and efficiency of annotation in future studies.

Whole-genome duplication events are widely present in plants, especially in Brassicaceae species [3, 6, 42, 64]. It has been reported that Brassicaceae experienced at least two specific whole-genome duplication events [50], while representatives of Brassicaceae Lineage II (including *Brassica* species) have also passed through whole-genome triplication [17, 41]. These events not only increase species diversity, but also enhance the adaptability to the environment [31]. *C. neglecta* has experienced α and β whole-genome duplication events, but no unique whole-genome duplication event.

The three sets of subgenomes of *C. sativa* have been previously distinguished [33] and later revised, based on wild *Camelina* species sequencing data [15]. This study used the assembled *C. neglecta* genome and calculated the Ks value of synteny gene pairs with *C. sativa* to successfully differentiate three sets of subgenomes in *C. sativa.* The present study confirmed that *C. neglecta* genome retains the highest similarity to SG1 of *C. sativa* and also shows high collinearity with SG2 of *C. sativa*. These findings are consistent with widely accepted hypothesis that *C. neglecta* could be the direct progenitor of SG1 of hexaploid *Camelina*, while SG2 may have originated from diploid *C. neglecta*-like progenitor, which has not been found yet or could be extinct [15, 16, 46, 48]. The origin of *C. neglecta* remains unclear, except the fact that this species faced chromosome number reduction, which led to diversification of *C. neglecta* from its possible *C. neglecta*-like ancestor with *n* = 7 chromosome counts [46]. In addition, *C. neglecta* retains the similar genome organization of the inferred ancestral *Camelina* genome [45, 46], except for the fact that two chromosomes are conjugated (chr1, in the present study).

Revealing the genome organization of *C. neglecta* further improves the understanding of the origin and evolutionary history of *C. sativa* and other related species. It has been reported that *C. sativa* might not have faced any post-polyploidization chromosome rearrangement events, which makes it a perfect model for studying the neo-ploidy in higher plants [15, 46, 48]. Moreover, it was shown that the direct progenitors of *C. sativa* also retain the subgenome structure of their parental species. In particular, *C. intermedia* (tetraploid *C. microcarpa*, 2*n* = 26) and hexaploid *C. microcarpa* (2*n* = 40, Type 1) show identical organization of chromosomal regions in SG1 and SG2 [45, 46]. On contrary, *C. rumelica*, other tetraploid representative of the genus, shows signs of significant chromosomal rearrangements within SG1, whose origin is associated with the *C. neglecta* [15, 46].

The origin of alternate cytotype of *C. microcarpa* (2*n* = 38, Type 2) remains a mystery. This cytotype is distinguished by a completely different organization of the third subgenome. The origin of SG3 in *C. sativa* is associated with its direct inheritance from *C. hispida* (possibly var. *hispida*) [46, 48]. At the same time the third subgenome in *C. microcarpa* Type 2 is associated with the *C. neglecta*-type genome, which might have different genome organization from the *C. neglecta*, but also had the reduced chromosome number [9, 15, 45]. Otherwise, this mysterious subgenome of *C. microcarpa* Type 2 could carry signs of significant chromosome structure rearrangements, if it was inherited directly from the common *C. neglecta*. Finally, it should be noted that *C. neglecta* was identified as the progenitor of a common cytoplasmic lineage of *C. intermedia*, both types of *C. microcarpa* and *C. sativa* [9, 45].

*C. neglecta* is known to contain a significant amount of unsaturated fatty acids, similarly to all characterized species of this genus [9]. By analyzing transcriptomic data, we discovered that some key genes involved in the oil biosynthesis pathway were highly expressed in seed. These genes include desaturase-coding genes *SAD*, *FAD2* and *FAD3*, along with the elongase-coding gene *FAE1*, which are involved in the biosynthesis of the major fatty acids in *Camelina* seed oil. We found that the highest expression of *CnFAD2* and *CnFAD3* was measured during the mid and late stages of seed development. The expression of *CnFAE1* was found to increase within the passage from early to late stages of seed development of *C. neglecta*. Additionally, several key transcription factors, such as *ABI3*, *WRI1* and *FUS,* were found to be expressed. Collectively, these expressed genes likely contribute to the high content of polyunsaturated fatty acid-rich oil in *C. neglecta* seed.

Notably, the subgenomes of *C. sativa* may differentially contribute to the total expression rates of homologous genes. Previous transcriptomic research revealed that, despite the general expressional dominance of SG3 at various developmental stages and in different tissues of *C. sativa*, the expressional activity of genes of SG1 rapidly increases during seed development [15]. This fact suggests that the genes of SG1 might have an increasingly important role in developing seeds of *C. sativa*. Moreover, the genes, arising from *C. neglecta*-like genomes could have a great impact on regulating *C. sativa* development, since SG1 and SG2 are highly collinear and both contribute 28–33% each to the total expression in *C. sativa* [15]. All these findings suggest the high representability of *C. neglecta*, if it is used as a model species for molecular genetic research, instead of *C. sativa*.

Considering the important role of *C. neglecta* in the origin of *C. sativa* and their high similarity in lipid biosynthesis pathway, here we for the first time propose to use this diploid species as a model for *Camelina* oilseed biotechnology. In particular, we successfully demonstrate the amenability of *C. neglecta* for transformation, aimed on introgression of the genes, leading to alteration of fatty acid composition in seed oil. It has been shown that *C. neglecta* transformants demonstrate the desired changes in accumulation of C10:0, which is naturally not present in seed oil of any *Camelina* species. The observed change in fatty acid composition of *C. neglecta* seed oil is consistent with our previous findings on *C. sativa* [29].

We also established that the differences in vegetation cycle of winter *C. neglecta* plants and spring *C. sativa* do not have crucial impact on the fatty acid composition of seed lipids in both species. In addition, the homozygosity of *C. neglecta* transformants can be achieved only within three generations, while already in T_3_ plants the desired changes in fatty acid composition may be observed. Moreover, hexaploid genome organization of *C. sativa* significantly complicates gene editing research, since many target genes are represented by homologous triplets. Because of this hexaploidy, additional efforts are needed to obtain and identify edits of all possible allelic variants of target genes and later leads to high rates of mosaicism in transformants [30]. The use of *C. neglecta* would lessen such complications arising from the use of a hexaploid species.

The *C. neglecta* genome provided here enriches the understanding of *Camelina* genus evolution and offers resources for the biosynthesis of unsaturated fatty acids. Furthermore, it will likely contribute to the study of functional genomics and genetic improvement of *Camelina* crops. Finally, *C. neglecta* can be used as an efficient and highly representative model for studying effects of genetic engineering or gene editing, aiming on improvement of various traits of *Camelina* species, including seed oil biosynthesis as well as input traits that promote environmental resilience.

## Materials and methods

### Plant materials and sequencing

*C. neglecta* line (PI650135) was obtained from USDA-NPGS Genbank. The same line was previously used for the species description [8]. Also *C. sativa* cv. Suneson was used in the experiments. *C. neglecta* and a set of *C. sativa* plants were grown via the described protocol, which includes vernalization step [47], while the part of *C. sativa* plants were cultivated in greenhouse as the regular spring plants, excluding the vernalization stage.

Young *C. neglecta* leaves were used to construct both Illumina paired-end libraries and PacBio SMRTbell libraries. For Illumina sequencing, Illumina NovaSeq system was used to generate high-throughput reads with a length of 150 bp according to the manufacturer’s instructions (Illumina). For PacBio sequencing, PacBio Sequel II was used to generate PacBio long reads and PacBio SMRTbell libraries (~ 20 kb) was constructed using SMRTbell Template Prep Kit 1.0 (PacBio, Menlo Park, CA) following standard manufacturer protocol. To generate transcriptome data, RNA was extract from early developing seed (7–9 DAF), mid developing seed (13–15 DAF), late developing seed (18–20 DAF), leaf and stem were used for transcriptomics analysis. Three biological replicates were performed for each tissue.

### Genome assembly and quality assessment

The *C. neglecta* genome was de novo assembled using Canu (v2.1) [35] based on PacBio long reads with the following parameters: -corOutCoverage 200 -correctedErrorRate 0.045 -minOverlapLength 500 –rawErrorRate 0.3 –batOptions -dg 3 -db 3 -dr 1 -ca 500 -cp 50. Then the raw contigs were polished using Racon [60] based on PacBio long reads and polished using Pilon [62] based on Illumina short reads. Finally, the polished contigs were anchored into chromosomes by Juicer [21] and 3D-DNA [20] based on Hi-C reads and then the assembled genome was manual corrected by Juicexbox (v1.11.08).

To assess the accuracy and completeness of the genome assembly, the Illumina pair-end reads were mapped to the assembled genome with BWA-MEM (https://github.com/lh3/bwa), and RNA sequencing data were also mapped to the assembly with HISAT2 [34]. Furthermore, the BUSCO [56] and LAI were also used to assess the completeness of the assembly based on Embryophyta Plant database (odb10) and LTR_retriver [53].

### Genome annotation

The annotation of transposable elements is divided into homology prediction and de novo prediction methods. RepeatModeler, LTR_FINDER [65], RepeatScount [55] were used to build a de novo transposable element (TE) database and then combine the Repbase database (http://www.girinst.org/repbase) [32] and the de novo transposable element database to generate a consensus library. Finally, RepeatMasker (https://www.repeatmasker.org/) was used to identify repeat sequences.

The annotation of coding gene structure is to combine homology-based prediction, de novo prediction and RNA-Seq assisted prediction. First, the non-redundant protein from four closely related species of *C. neglecta* (*A. thaliana*, *A. lyrata*, *C. sativa*, *B. oleracea*, *B. napus*) were used as homology-annotation library and input for TBLASTN to search for homologous sequences. Furthermore, Augustus [58], Genscan [11] and GilmmerHmm [43] were used for de novo gene prediction. Third, RNA sequencing data were mapped to the genome using HISAT2 [34] and StringTie pipeline [54] was used for transcripts assembly. Finally, MAKER [27] and HiCESAP were used to combine all the predicted results to get a non-redundant gene set. In addition, the gene were functionally annotated with the help of protein databases SwissProt (https://www.gpmaw.com/html/swiss-prot.html), TrEMBL [2], NR (https://ftp.ncbi.nlm.nih.gov/blast/db/FASTA/), KEGG (https://www.genome.jp/kegg/) [52], InterPro [70] and GO [1].

The annotation of non-coding RNA: tRNAscan-SE [14] was used to annotate tRNA according to the structure characteristics of tRNA. Annotation of rRNA was based on mapping rRNA sequences to the genomes of closely related species using BLASTN. In addition, using the covariance model of the Rfam family, the INFERNAL with Rfam was used to predict the miRNA and snRNA.

### Phylogenetic analysis and gene family expansion/contraction

We selected *A. trichopoda*, *A. thaliana*, *B. oleracea*, *Beta vulgaris*, *C. rubella*, *Glycine max*, *J. curcas*, *Oryza sativa*, *Populus trichocarpa*, *R. communis*, *Solanum lycopersicum*, *Sorghum bicolor*, *Theobroma cacao*, *V. vinifera*, *Zea mays* to investigate the evolutionary of *C. neglecta* and their genomes were downloaded from Phytozome database (https://phytozome-next.jgi.doe.gov/) [24]. First, a total of 174 sing-copy orthologous genes were identified with Orthofinder [23] and the protein sequences and coding sequence of these gene were subject to multiple sequence homology alignment with MUSCLE [22]. Then ProtTest [18] was used to predict the best-fit model (JTT+I+G+F) for constructing the phylogenetic tree. According to the result of ProtTest, RAxML [57] was used to construct the phylogenetic tree. Finally, the iTOL website (https://itol.embl.de/) [37] was used to visualize the phylogenetic tree.

MCMCTree program in the PAML package (v.4.9.j) [66] was used to calculate the divergence time for the above 16 species. The calibration points used in the MCMCTree were from the TimeTree database (http://www.timetree.org/) [36] and *A. thaliana*–*V. vinifera* split time (mean time 117 MYA), *A. thaliana*–*B. oleracea* split time (mean time 26.0 MYA), *O. sativa*–*Z. mays* split time (mean time 49 MYA) were chosen. Finally, the gene family expansion and contraction were calculated by CAFÉ [19] based on the phylogenetic tree and the number of gene families.

### Genome comparative analysis

OrthoMCL [38] was used to calculate the number of gene families and *C. neglecta*, *C. rubella*, *J. curcas*, *R. communis* and *B. oleracea* were selected for analysis. MCScan (python version) [https://github.com/tanghaibao/jcvi/wiki/MCscan-(Python-version)] was used to find pairwise genes and multiple genome syntenic comparisons and visualizations. First, MCScan was used to search pairwise genes of the *C. neglecta* and *C. sativa* and KaKs_calcutator [63] was used to calculate the Ks of these pairwise genes. Secondly, MCScan was used to visualize the synteny of the *C. neglecta* and *C. sativa* and the Ks of the pairwise genes.

### Genetic transformation of C. neglecta

A binary vector, expressing *CvFatB1* gene (from *Cuphea viscosissima*), under seed-specific glycinin promoter [29, 59], was modified for the present study. To enhance efficiency of medium-chain fatty acids (C10:0–14:0) accumulation, following seed-specific transgenes were introduced into the vector: lysophosphatidic acid acyltransferase (LPAT), diacylglycerol acyltransferase (DGAT) from *Cuphea* species. In particular, *CvLPAT2* (from *C. viscosissima*) was set under oleosin promoter, while *CpDGAT1* (from *Cuphea avigera* var. *pulcherrima*) was under the control of glycinin-1 seed-specific promoter, while *CvFatB1* coding sequence was under the control of Glycinin-1 promoter (Additional file 1: Fig. S9). The backbone of the vector is derived from pCAMBIA0380 and was engineered with the DsRed marker gene under the control of the constitutively expressed cassava mosaic virus promoter [29, 51] for selection of transgenic seeds by fluorescence [40].

*C. neglecta* plants, used for transformation, were grown similarly as described above under greenhouse conditions. *Agrobacterium tumefaciens* cells (strain C58C1) with the binary vectors containing *FatB* cDNAs were transformed by electroporation. Camelina plants were transformed in planta by floral dip/vacuum infiltration, and DsRed was used as a visual marker for selection [40].

### Determination of fatty acid composition and seed oil content

Fatty acid methyl esters (FAMEs) were prepared via transesterification, using trimethylsulphonium hydroxide [12]. About 12–15 mg seeds (three biological replicates) were directly crushed in 50 µL of TMSH in glass GC vials. Heptane (400 µL) was added to each vial. After room temperature incubation with agitation for 30 min, FAMEs were analyzed by gas chromatography as described previously [13]. Total fatty acid content of seeds was measured using C17:0-TAG as an internal standard for gas chromatography-flame ionization detection analysis using transesterification and extraction methods as previously described [13]. Statistical analysis of fatty acids content in seeds of *C. neglecta* and *C. sativa* was performed according to the previously described procedure [4].

### Supplementary Information

**Additional file 1: Fig**. **S1.** Genome size estimation of *C. neglecta* genome using k-mer analysis with different k-mer lengths. (a) 17mer (b) 19-mer (c) 21-mer (d) 23-mer. **Fig. S2.** Divided collinear synteny blocks into sub1, sub2 and sub3 from top to bottom by each chromosome of *C. neglecta.* **Fig. S3.** The Ks distribution of the collinear synteny blocks in each chromosome of *C. neglecta.* **Fig. S4.** The synteny plot of this study and the two published *C. neglecta* genomes. **Fig. S5.** The collinearity of three *C. neglecta* genomes. **Fig. S6.** PacBio reads coverage at the inversion breakpoints assembled by Chaudhary et al. and our assembly on chromosome 2. **Fig. S7.** The Hi-C signal heatmap of the 4.2 Mb inversion region on chromosome 2 (extending 2.5 Mb on the left and right sides of the inversion region). **Fig. S8.** Verified the accuracy of the 9 large presence variations in the genome we assembled. **Fig. S9.** Binary vector, used for *Agrobacterium*-mediated *in planta* transformation of *C. neglecta*.

**Additional file 2: Table S1.** Raw sequencing data. **Table S2.** Anchored chromosome length of *C. neglecta*. **Table S3.** Evaluation of *C. neglecta* genome based on RNA-seq of different tissues. **Table S4.** Evaluation of assembly completeness. **Table S5.** Genetic structure of *C. neglecta*. **Table S6.** Gene function annotation statistics. **Table S7.** Statistics of transposable element of *C. neglecta*. **Table S8.** LTR insertion time in *C. neglecta* genome. **Table S9.** Statistics of annotated non-coding RNAs. **Table S10.** The GO enrichment analysis of unique gene families of *C. neglecta*. **Table S11.** The key genes involved in the oil biosynthesis pathway and their TPM in *C. neglecta*. **Table S12.** Comparison with published *C. neglecta* assemblies. **Table S13.** Gene annotation quality statistics.

## Data Availability

The *C. neglecta* genome assembly has been deposited in the Genome Warehouse in National Genomics Data Center (Members and Partners 2023), Beijing Institute of Genomics, Chinese Academy of Sciences/China National Center for Bioinformation, under Bioproject number PRJCA016289 that is publicly accessible at https://ngdc.cncb.ac.cn/gwh.
