# Supplementary material for: Chromosome-level assembly and analysis of Camelina neglecta: a novel diploid model for Camelina biotechnology research
Source: Biotechnol Biofuels Bioprod. 2024 Jan 31;17:17. doi: 10.1186/s13068-024-02466-9 (PMC10829252; doi:10.1186/s13068-024-02466-9)
Supplement: Supplementary file 1 — Additional file 1: Fig. S1. Genome size estimation of C. neglecta genome using k-mer analysis with different k-mer lengths. (a) 17mer (b) 19-mer (c) 21-mer (d) 23-mer. Fig. S2. Divided collinear synteny blocks into sub1, sub2 and sub3 from top to bottom by each chromosome of C. neglecta. Fig. S3. The Ks distribution of the collinear synteny blocks in each chromosome of C. neglecta. Fig. S4. The synteny plot of this study and the two published C. neglecta genomes. Fig. S5. The collinearity of three C. neglecta genomes. Fig. S6. PacBio reads coverage at the inversion breakpoints assembled by Chaudhary et al. and our assembly on chromosome 2. Fig. S7. The Hi-C signal heatmap of the 4.2 Mb inversion region on chromosome 2 (extending 2.5 Mb on the left and right sides of the inversion region). Fig. S8. Verified the accuracy of the 9 large presence variations in the genome we assembled. Fig. S9. Binary vector, used for Agrobacterium-mediated in planta transformation of C. neglecta. [file 13068_2024_2466_MOESM1_ESM.pdf]

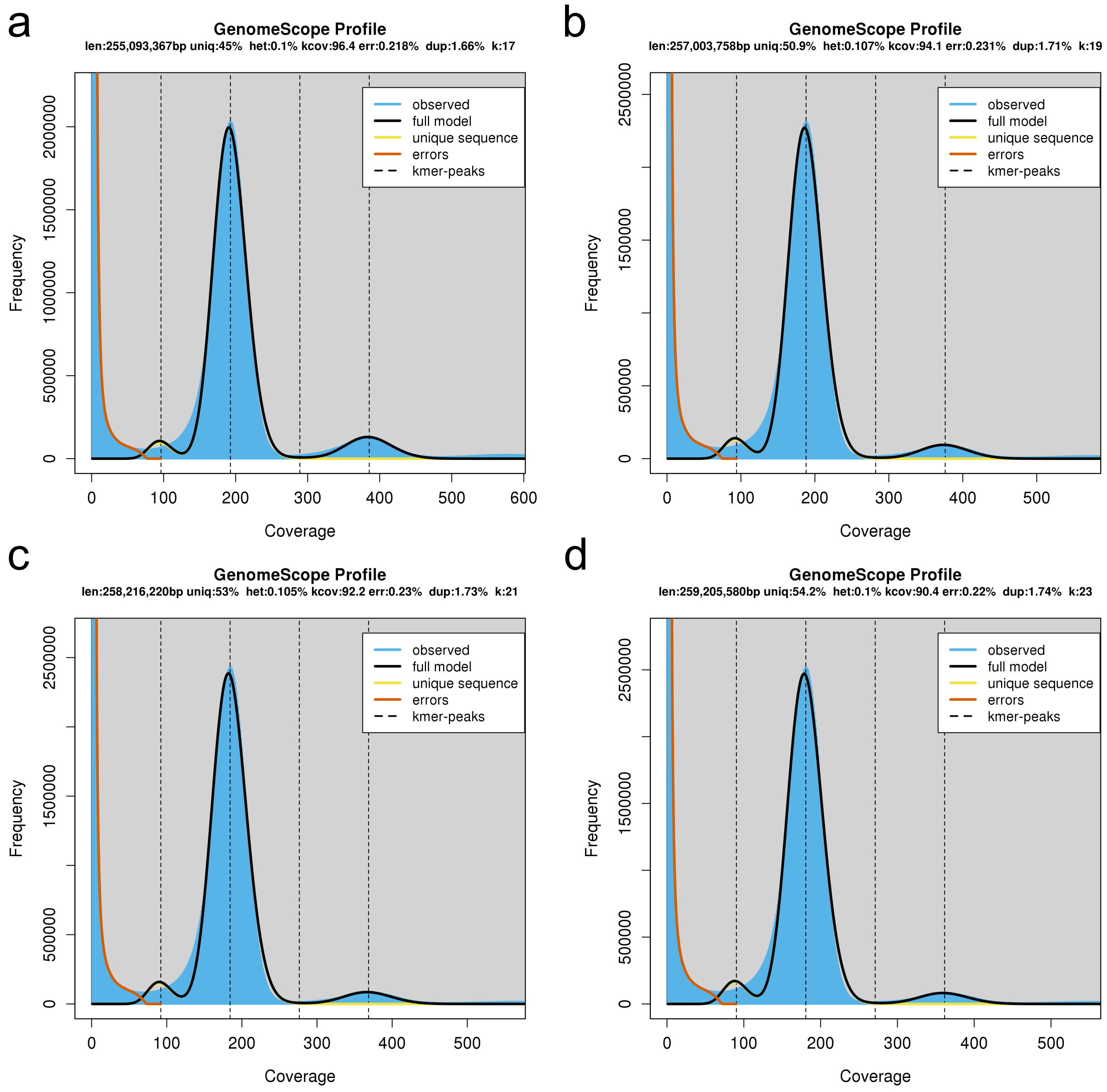

Fig. S1. Genome size estimation of *C. neglecta* genome using k-mer analysis with different k-mer lengths. (a) 17mer (b) 19-mer (c) 21-mer (d) 23-mer.

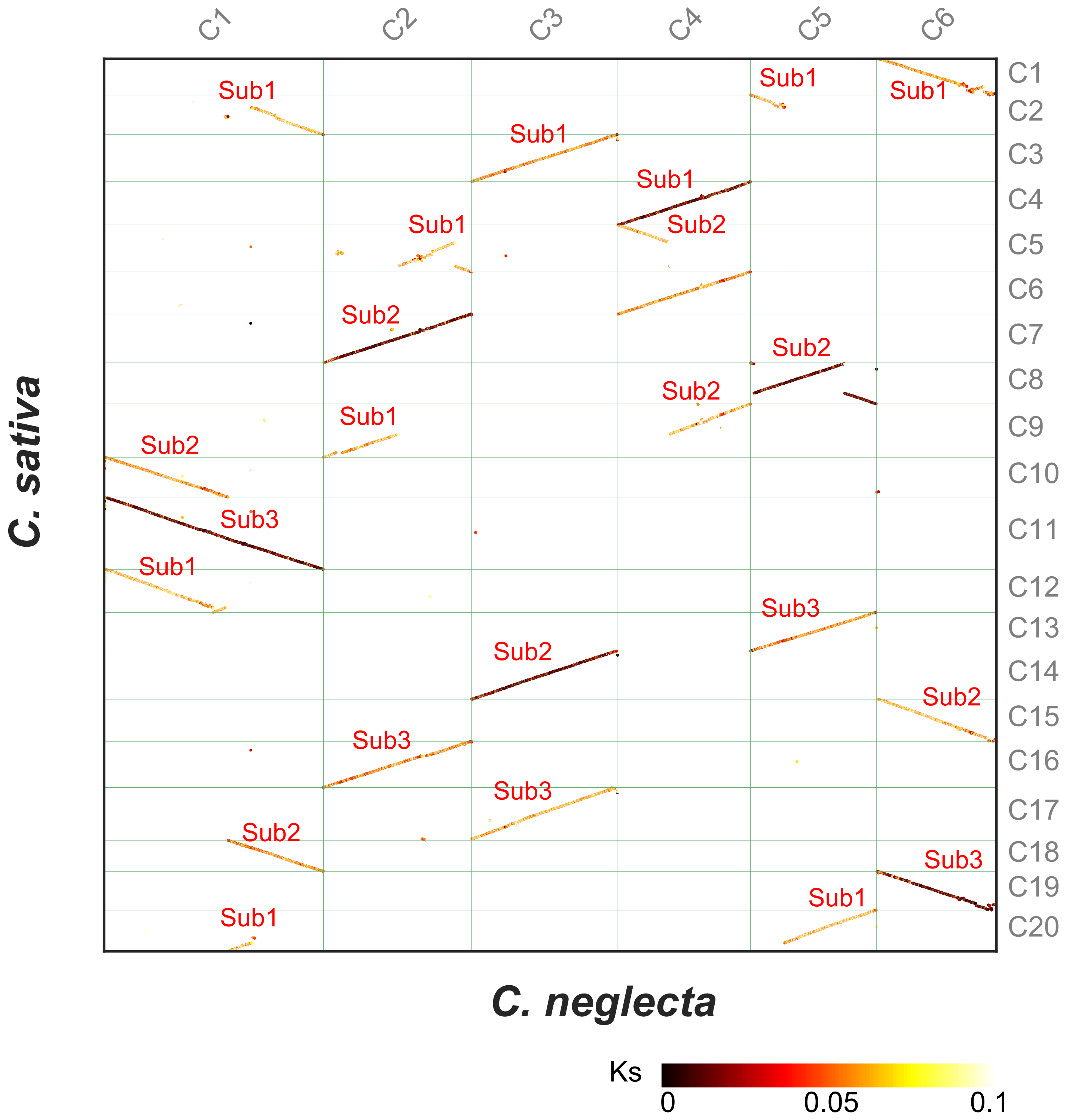

Fig. S2. Divided colinear synteny blocks into sub1, sub2 and sub3 from top to bottom by each chromosome of *C. neglecta.*

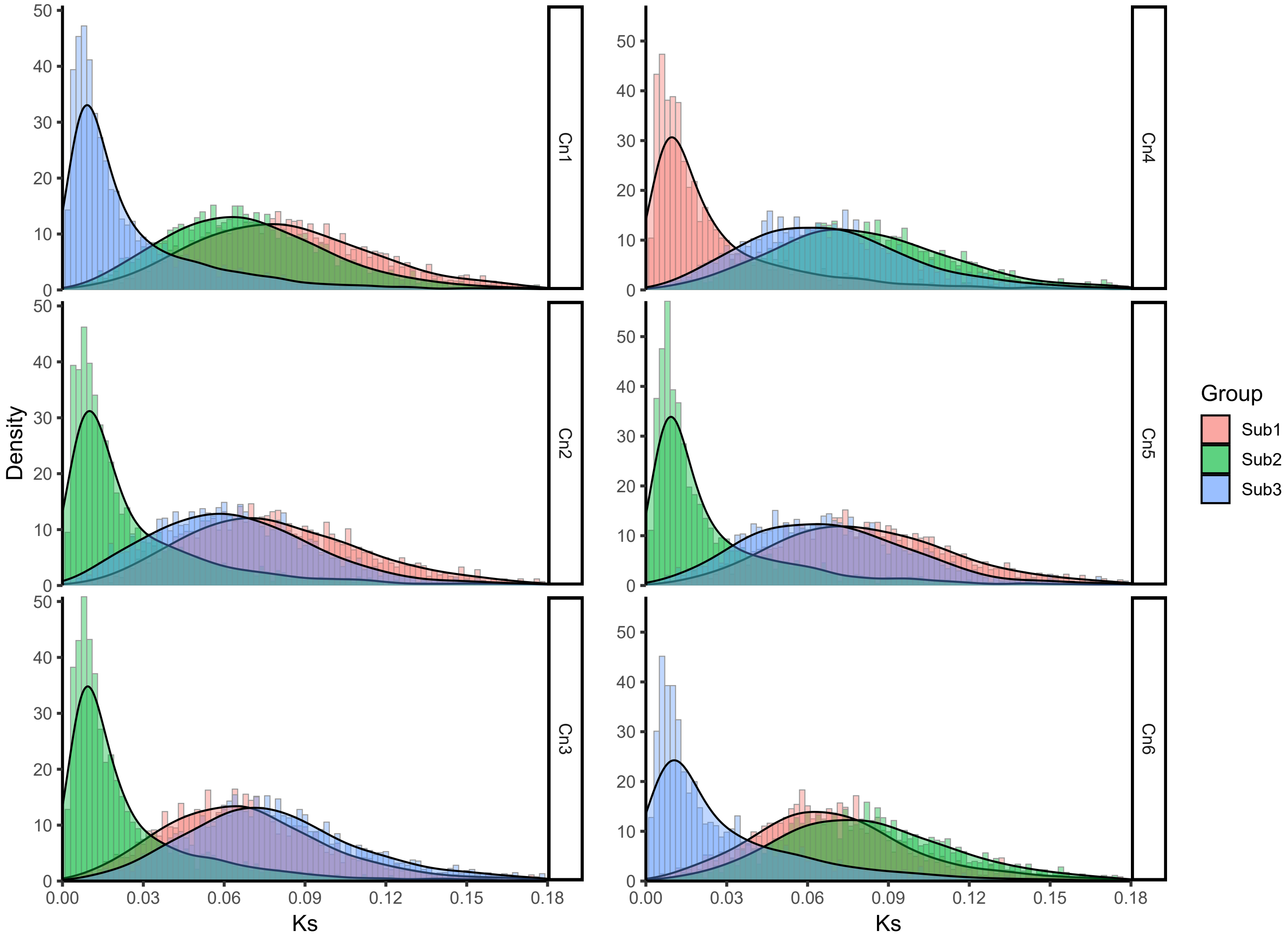

Fig. S3. The Ks distribution of the colinear synteny blocks in each chromosome of *C. neglecta.*

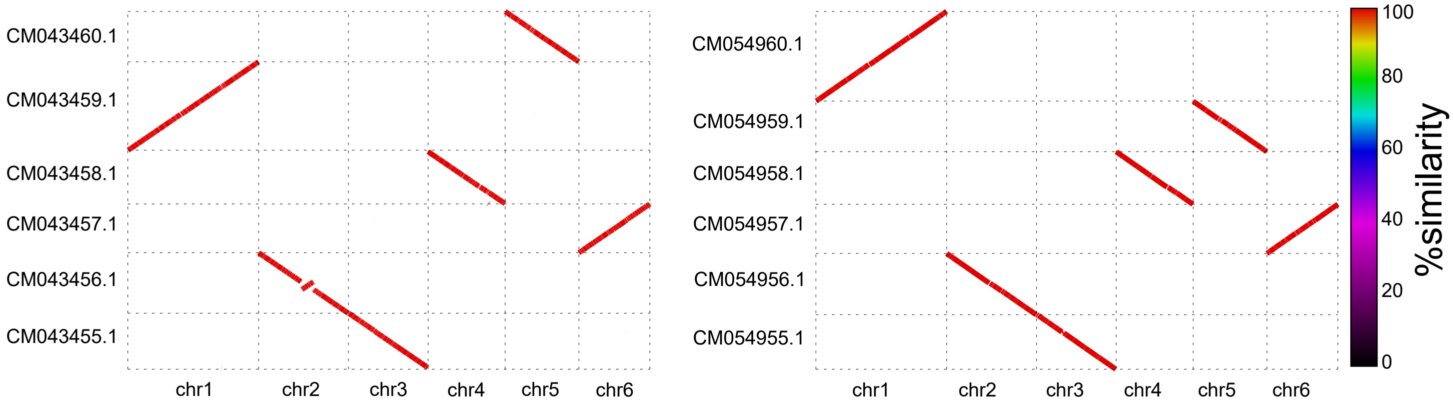

Fig. S4. The synteny plot of this study and the two published *C. neglecta* genomes. On the left, the horizontal direction is the genome of this study, the vertical direction is the assembly of Martin et al., There There was a 4.2 Mb inversion on chromosome 2 (Martin et al., 2022). On the right, the horizontal direction is the genome of this study, and the vertical direction is the assembly of Chaudhary et al (Chaudhary et al., 2023).

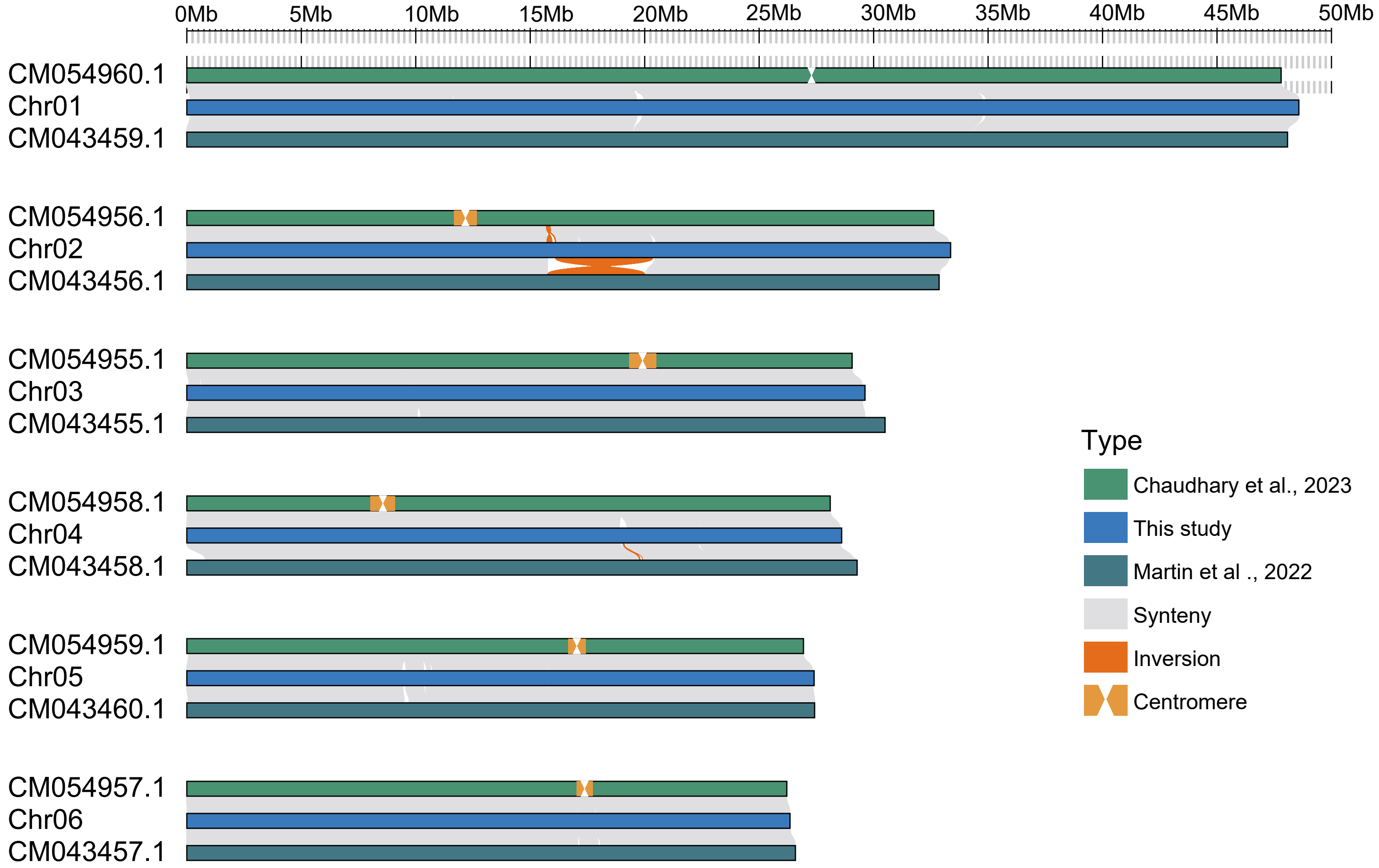

Fig. S5. The collinearity of three *C. neglecta* genomes.

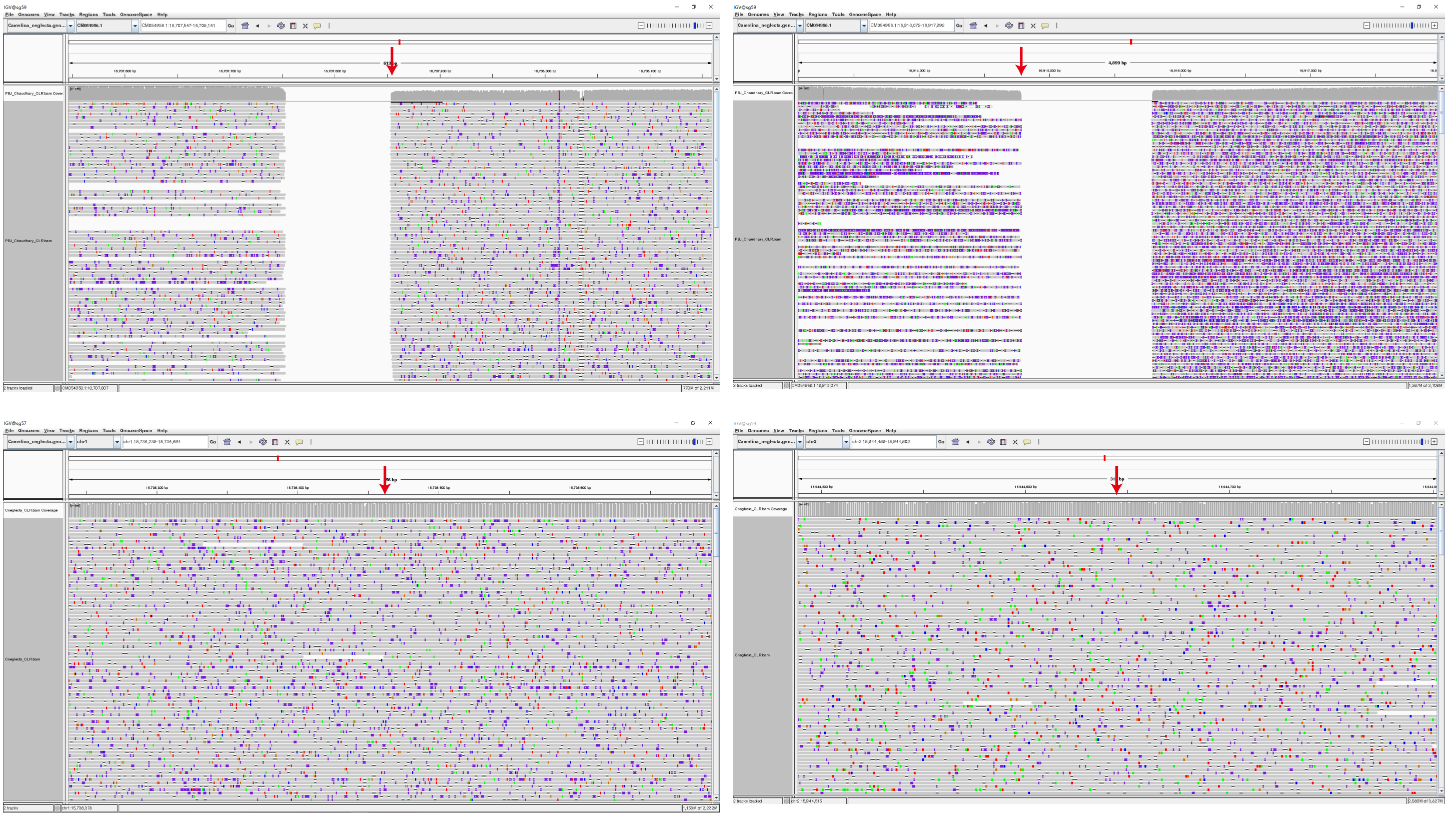

Fig. S6. PacBio reads coverage at the inversion breakpoints assembled by Chaudhary et al. and our assembly on chromosome 2. The coordinates of the breakpoints are as follows: CM054956.1:16707854 (upper left), CM054956.1:16914787 (upper right), chr2:15736466 (lower left), and chr2:15944646 (lower right) (Chaudhary et al., 2023).

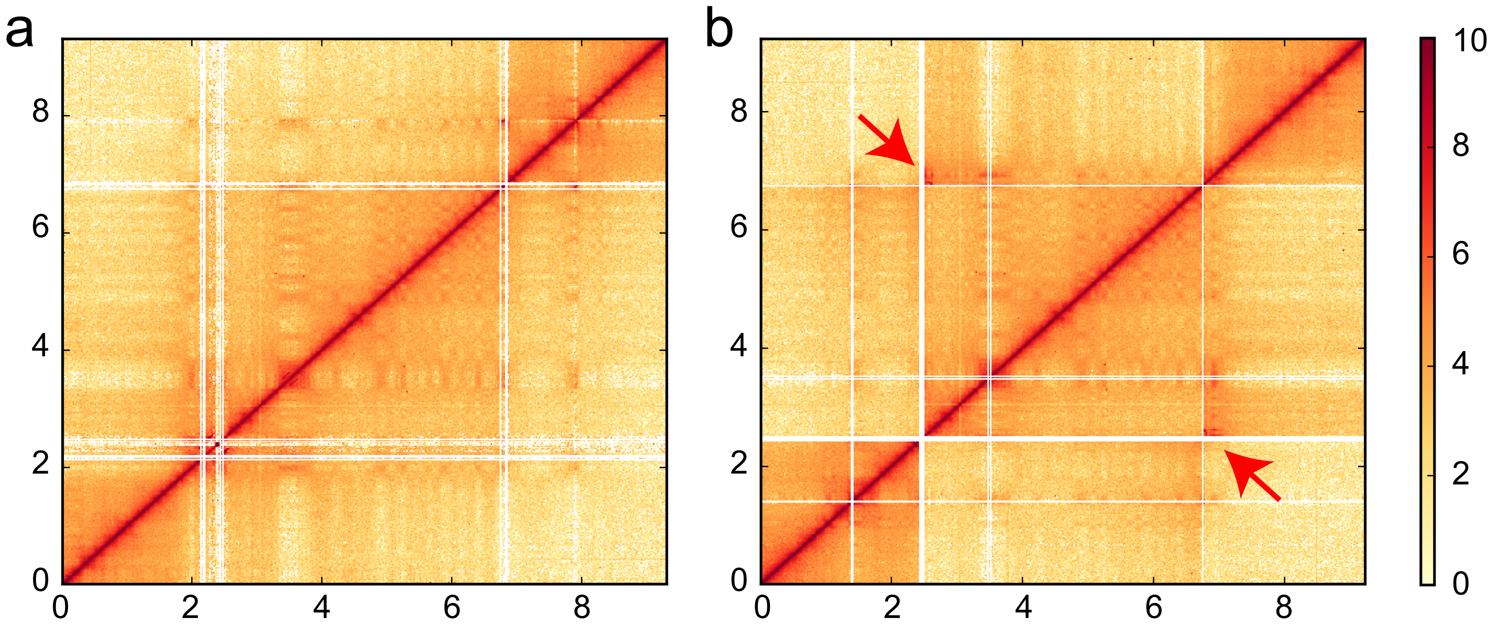

Fig. S7. The Hi-C signal heatmap of the 4.2 Mb inversion region on chromosome 2 (extending 2.5 Mb on the left and right sides of the inversion region). **(a)** The heatmap displayed the Hi-C signal of this region assembled in this study. **(b)** The Hi-C signal of this region assembled by Martin et al (Martin et al., 2022).

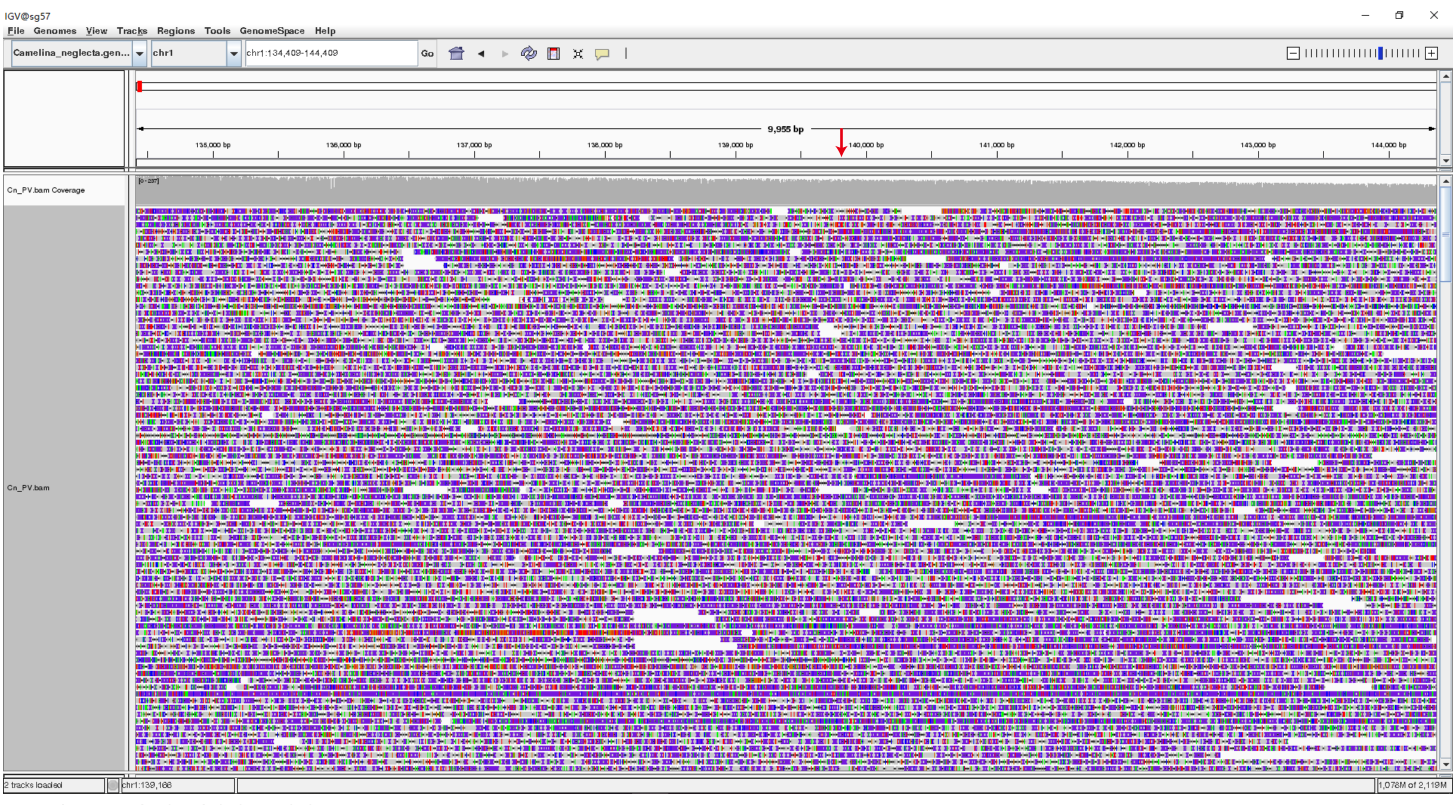

PV1 chr1:0-139,768

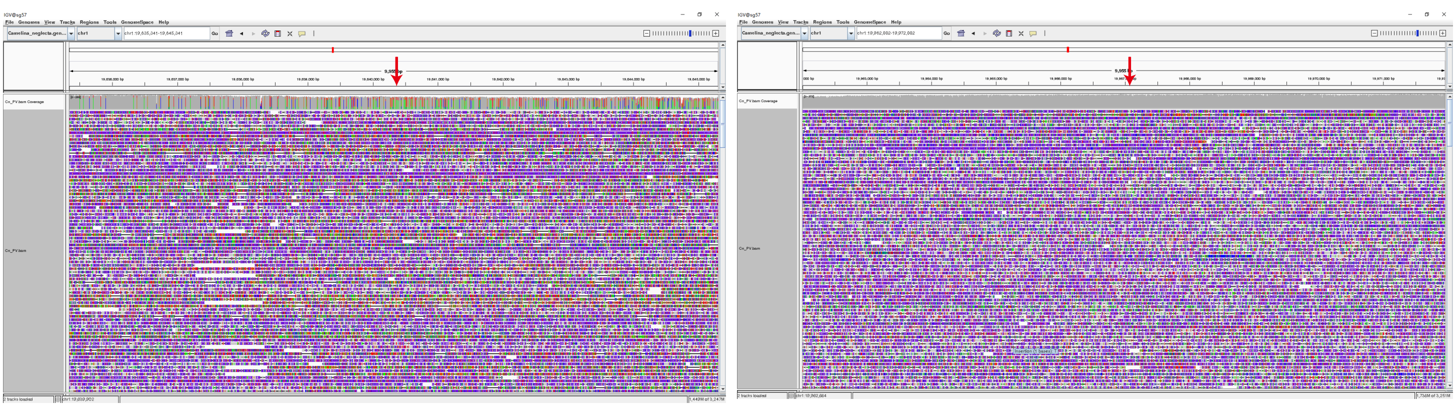

PV2 chr1:19,640,341-19,967,002

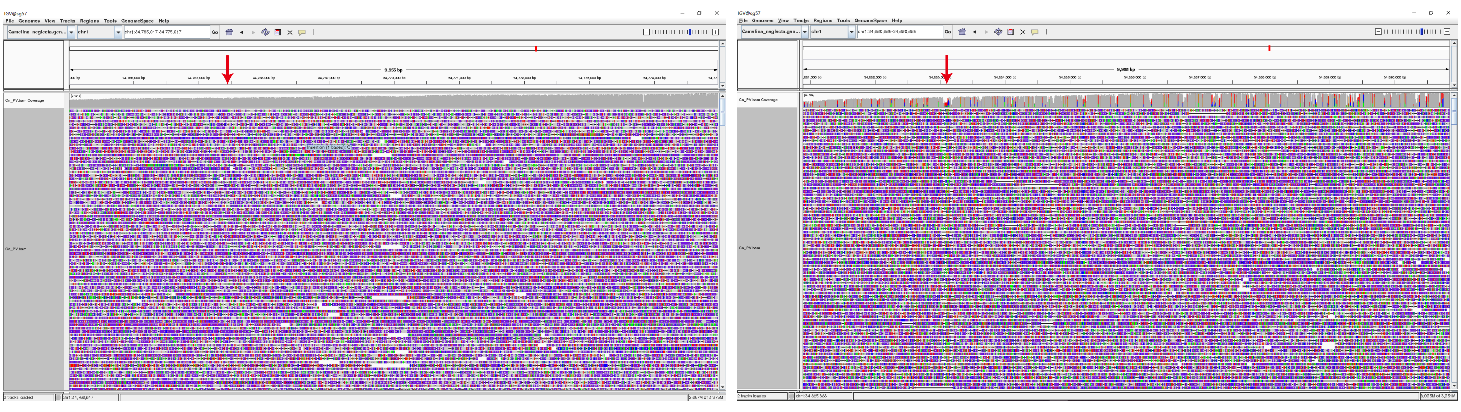

PV3 chr1:34,767,467-34,883,185

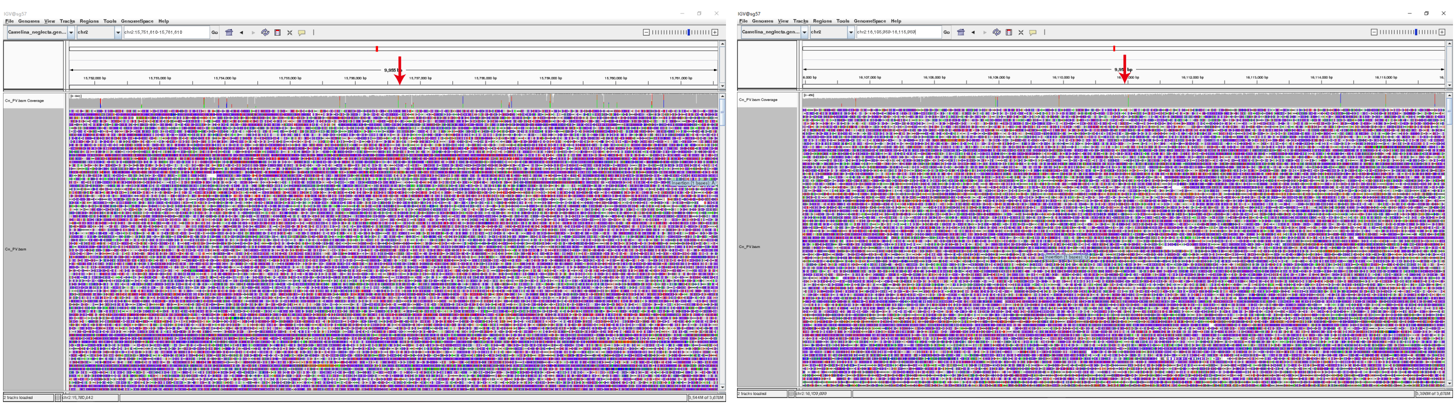

PV4 chr2:15,756,610-16,110,959

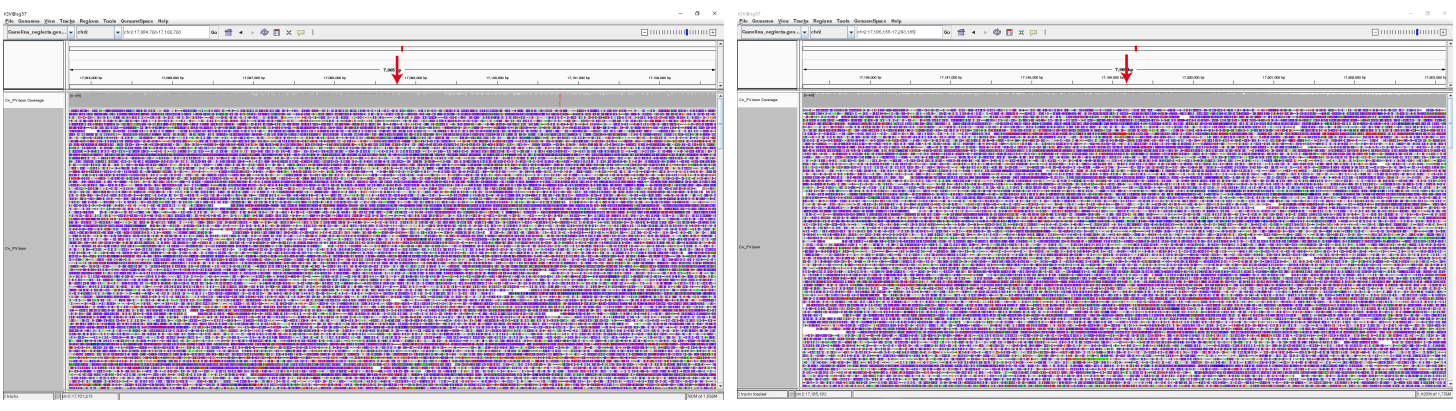

PV5 chr2:17,098,728-17,199,165

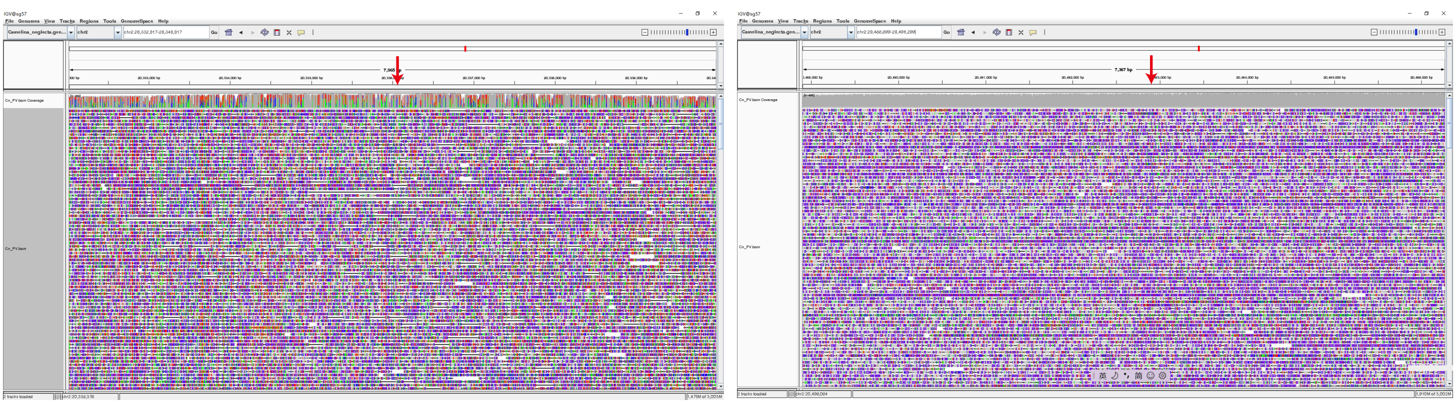

PV6 chr2:20,336,017-20,492,899

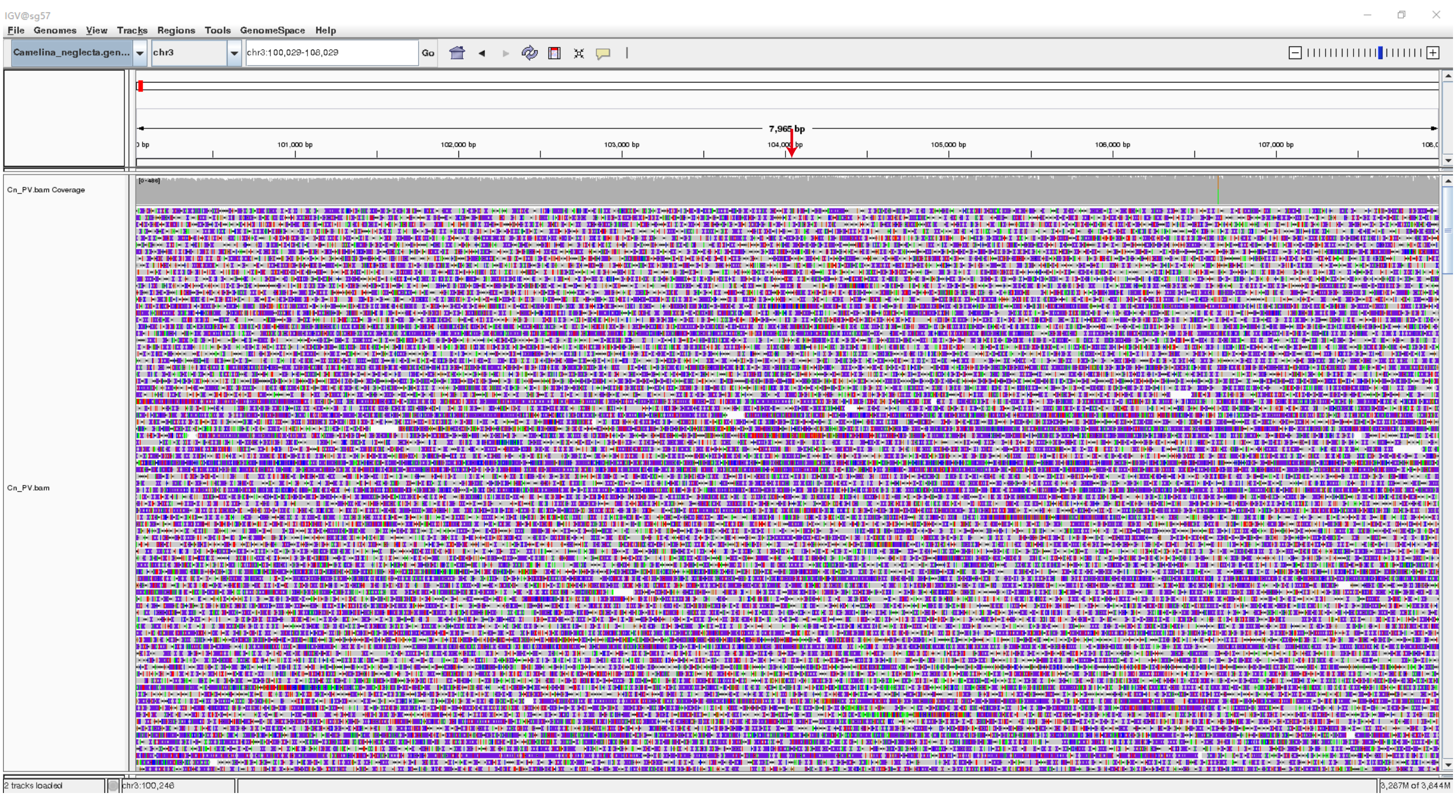

PV7 chr3:0-104,029

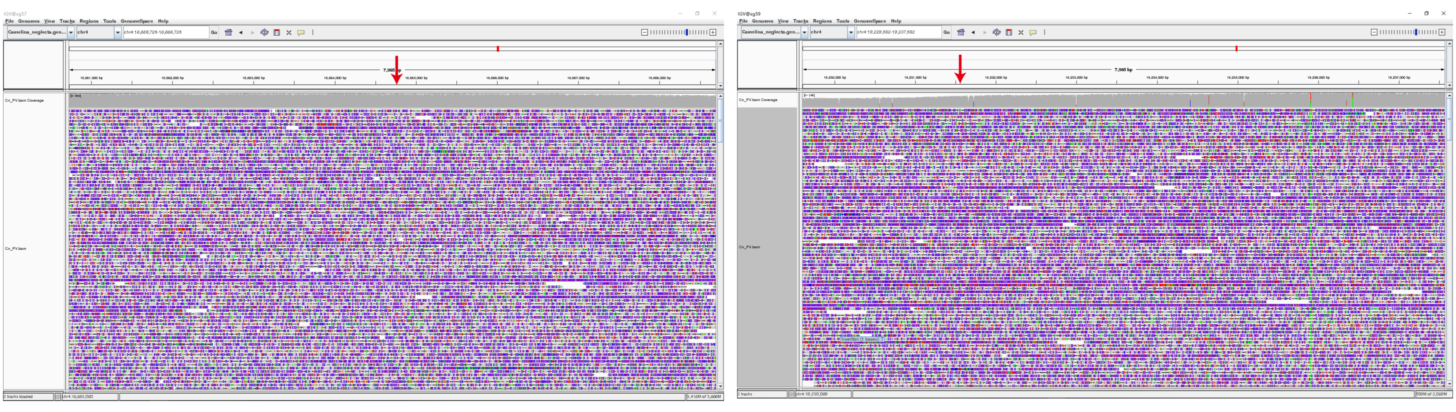

PV8 chr4:18,884,726-19,231,608

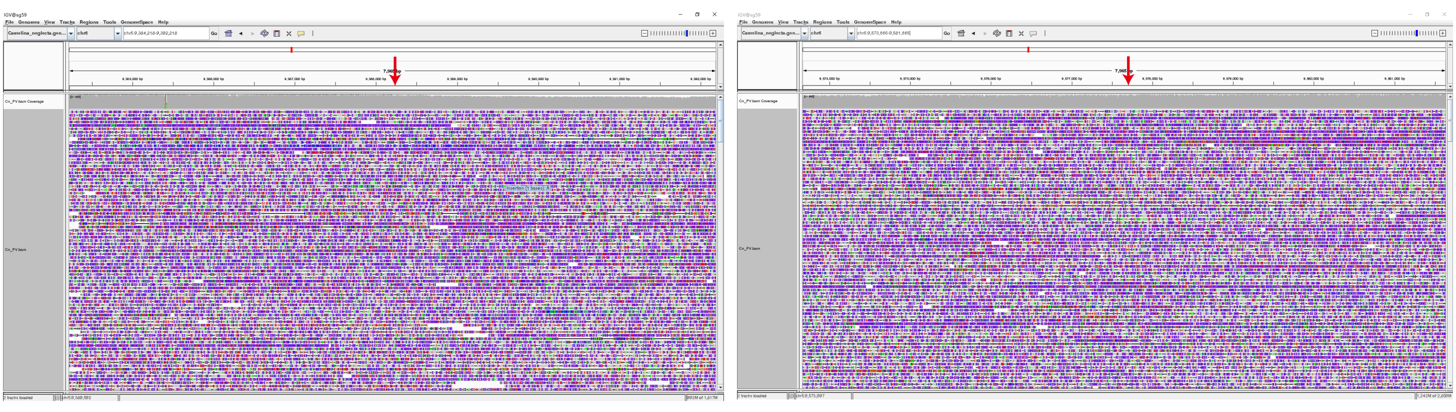

PV9 chr5:9,388,218-9,577,665

Fig. S8. Verified the accuracy of the 9 large presence variations in the genome we assembled. The PacBio read coverage with breakpoints at both ends of each variations is displayed. Variants located at the beginning or end are only displayed with a single breakpoint.

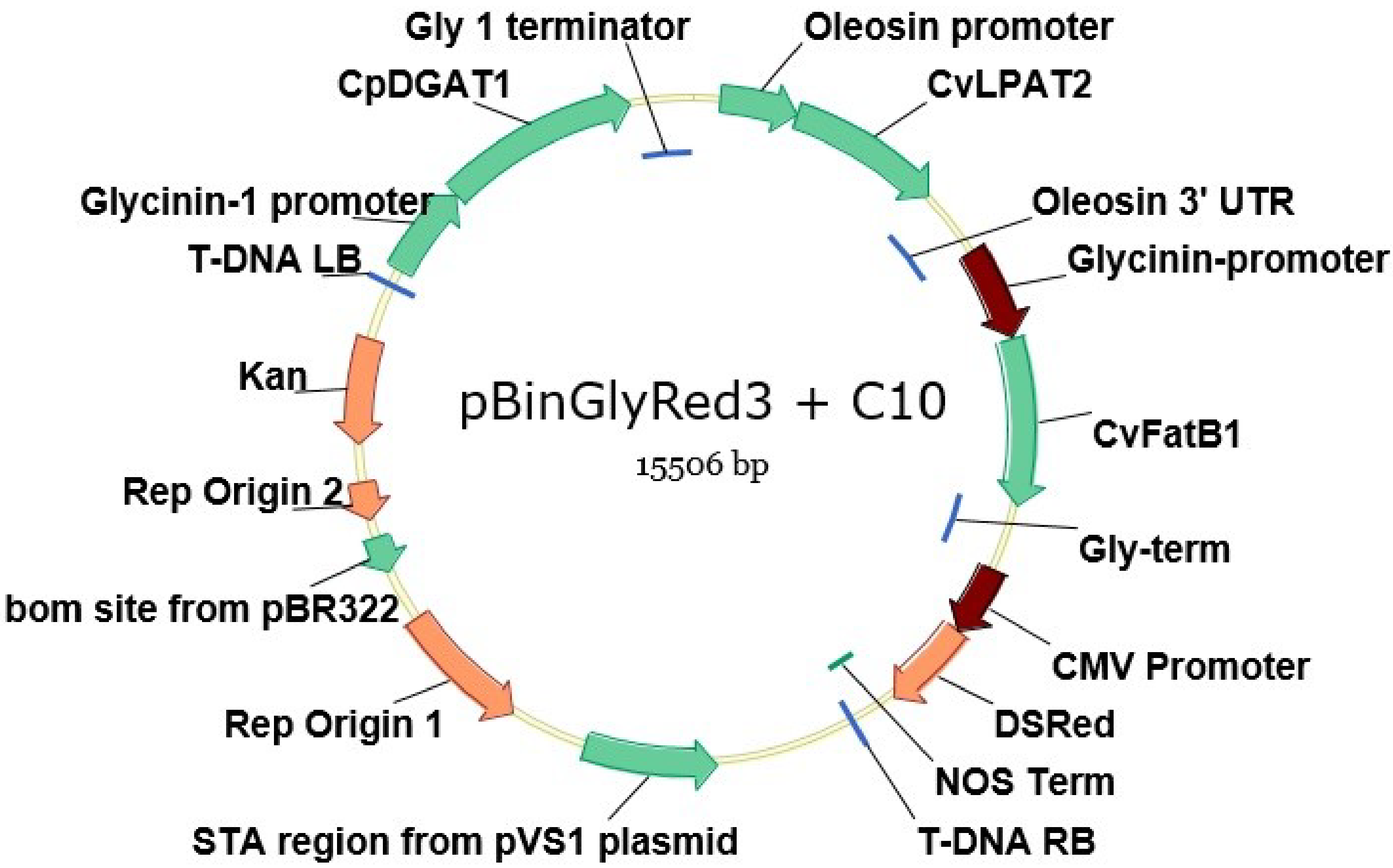

Fig. S9. Binary vector, used for *Agrobacterium*-mediated *in planta* transformation of *C. neglecta*.
